# Intestinal immunopathological evaluation of mice reinfected with *Trypanosoma cruzi* Y strain

**DOI:** 10.1371/journal.pntd.0013826

**Published:** 2025-12-15

**Authors:** Arthur Wilson Florêncio da Costa, Rafael Obata Trevisan, José Rodrigues do Carmo Neto, Yarlla Loyane Lira Braga, Anna Victória Bernardes e Borges, Ana Luisa Monteiro dos Santos Martins, Marlene Antônia dos Reis, Flávia Aparecida de Oliveira, Marcos Vinícius da Silva, Mara Rúbia Nunes Celes, Juliana Reis Machado

**Affiliations:** 1 Department of Bioscience and Technology, Institute of Tropical Pathology and Public Health, Federal University of Goiás, Goiania, Goiás, Brazil; 2 Discipline of General Pathology, Institute of Biological and Natural Sciences of Federal University of Triângulo Mineiro, Uberaba, Minas Gerais, Brazil; 3 Department of Microbiology, Immunology and Parasitology, Institute of Biological and Natural Sciences of Federal University of Triângulo Mineiro, Uberaba, Minas Gerais, Brazil; Advanced Centre for Chronic and Rare Diseases, INDIA

## Abstract

**Background:**

Chagas disease, caused by the protozoan *Trypanosoma cruzi*, is influenced by both host-related factors and parasite characteristics, including reinfection and strain variability. While the heart is the primary focus of reinfection studies, the effects on the intestinal form of the disease remain poorly understood.

**Methodology/Principal Findings:**

In this study, C57BL/6 male mice were infected and subsequently reinfected with the *T. cruzi* Y strain to assess the immunopathological consequences in the intestinal tract. We analyzed parasitemia levels, systemic cytokines, anti-*T. cruzi* immunoglobulin levels (IgG, IgG2a, IgG2b), inflammatory infiltrates, neuronal counts, and collagen deposition. Our data showed that reinfection led to reduced parasitemia and did not alter antibody levels generated during primary infection. However, reinfection induced a systemic reduction in IL-4 and IL-10 and an increase in IFN-γ, indicating a shift toward a pro-inflammatory profile. Histopathologically, reinfected mice exhibited intensified intestinal inflammation and increased neuronal destruction in the myenteric plexus, without additional collagen deposition compared to singly infected animals.

**Conclusions/Significance:**

Although homologous reinfection may enhance parasitemia control through sustained immunoglobulin responses, it exacerbates tissue inflammation and neuronal damage. These findings underscore the dual role of immune responses in controlling parasite burden while potentially contributing to intestinal pathology, highlighting the need for caution when considering reinfection risks in endemic areas.

## Author summary

Chagas disease, caused by the parasite *Trypanosoma cruzi*, affects millions of people in Latin America and can lead to severe damage in the heart and digestive system. In regions where the disease is common, people may be exposed to the parasite more than once during their lifetime, but the effects of reinfection on the digestive form of the disease are poorly understood. In this study, we used a mouse model to evaluate how reinfection with the same strain of *T. cruzi* affects the intestine. We found that reinfection helped control the number of parasites in the bloodstream, likely due to the persistence of specific antibodies. However, despite this protection, reinfected mice showed worse intestinal inflammation and more nerve damage in the intestinal wall. These findings suggest that reinfection may enhance the immune system’s ability to fight the parasite but at the cost of greater tissue damage. Understanding this dual effect is important for developing strategies to protect people living in areas where exposure to *T. cruzi* is frequent, and it may help explain how the digestive complications of Chagas disease develop over time.

## 1. Introduction

*Trypanosoma cruzi* (Kinetoplastida: Trypanosomatidae) is the causative agent of Chagas disease (CD), with insects of the genus Triatominae serving as potential vectors [1]. This parasite primarily infects populations residing in tropicais countries such as Brazil, Argentina, and Bolivia. Individuals living in precarious conditions and/or consuming food contaminated with feces of infected insects are more likely to acquire the infection [2]. First described by Carlos Chagas in 1909, CD is currently present in 21 Latin American countries and affects approximately 6 to 7 million people worldwide [3].

Infected individuals and residents of endemic areas are particularly susceptible to new exposures to *T. cruzi* [4–6]. Repeated exposures to the parasite have been reported to worsen clinical outcomes and contribute to the development of more severe forms of the disease [5,7–10]. Additionally, the specific strain of *T. cruzi* involved in infection plays a significant role in the disease’s progression. The parasite displays considerable genetic diversity among strains and/or clones, each associated with different biological behaviors in both humans and experimental models [5,6]. The heart and gastrointestinal tract are the most commonly affected organs [11–13].

The digestive form of the disease is characterized by the formation of megaviscera, marked by the dilation of organs such as the esophagus, stomach, and colon [14]. Human studies have shown that the development of megaviscera is associated with a reduction in neuronal density within the gastrointestinal tract and an increased intensity of local inflammatory responses [15,16]. Similarly, experimental models have demonstrated that *T. cruzi* infection is linked to intestinal inflammation and a reduction in the neuronal density of the myenteric plexus, leading to impaired intestinal motility and function [17,18].

Most studies involving reinfection are experimental and typically focus on parameters such as parasitemia, survival, and tissue parasitism [19–23]. The heart remains the most extensively studied organ in this context, while relatively few investigations have addressed intestinal histopathological alterations following *T. cruzi* reinfection [5,7,10,24–27]. Therefore, the aim of this study was to evaluate the development of intestinal morphological lesions and the systemic immune response in male C57BL/6 mice infected and reinfected with the Y strain of *T. cruzi*.

## 2. Methods

### 2.1. Ethics statement

Approved by the Animal Ethics Committee at the Federal University of Goiás (Protocol: 051/19).

### 2.2. Animals

In the present work, C57BL/6 male mice, weighing 22–27 g, were used. All animals were provided by the *bioterium* from Instituto de Patologia Tropical e Saúde Pública at Universidade Federal de Goiás (IPTSP/UFG) and maintained in a special room, with constant air renewal, at a temperature between 20 and 25ºC. and humidity between 45 and 55%. Feeding and drinking water were offered *ad libtium*. The research project was approved by the Ethics Committee on Animal Use of the Federal University of Goiás (Comitê de Ética no Uso de Animais da Universidade Federal de Goiás – CEUA/UFG), under protocol number 051/19.

### 2.3. Infection, parasitemia and euthanasia

The study included 17 animals divided into three groups: an uninfected control group (Control; n = 5), a group infected only with Y strain without reinfection (Y; n = 6), and a group infected and reinfected with Y strain (Y-Y; n = 6). All animals were infected and reinfected with 1,000 *T. cruzi* trypomastigote forms subcutaneously. The Y strain was chosen for primary infection due to its classical intestinal immunopathological profile [28–30].

On day 90 post-infection, animals from the Control and Y groups were euthanized, while animals from the Y-Y group were euthanized on day 108 (18 days after reinfection), following an established protocol. Animals were anesthetized with intraperitoneal Xylazine and Ketamine (50 mg/kg), and euthanized by cervical dislocation. Blood and intestinal samples were collected and stored at -80°C.

Parasitemia was monitored every 3 days for 30 days (initial infection), totaling 10 counts. Following reinfection (day 90), parasitemia was assessed again every 3 days for 18 days (6 counts). For each evaluation, 5 μL of blood was collected from the tail vein, placed between slide and coverslip, and 50 random fields were examined under a light microscope to count circulating trypomastigotes.

### 2.4. Preparation of antigens from *Trypanosoma cruzi* for immunoglobulin measurement

Soluble antigens of *T. cruzi* were obtained from cultures of epimastigote forms of *T. cruzi* in the stationary growth phase, cultivated in Schneider medium supplemented with 20% fetal bovine serum. The parasite suspension was washed three times by centrifugation at 600xg for 30 minutes at 4°C in phosphate-buffered saline (PBS). After the last centrifugation, 10⁸ epimastigote forms were resuspended in 1 ml of lysis buffer containing *N*_α_-Tosyl-L-lysine chloromethyl ketone hydrochloride (TLCK) and kept on ice under agitation for 10 minutes. The suspension was centrifuged at 4°C at 10,000 x g. Subsequently, the supernatant was collected, filtered through a membrane with a pore size of 0.22 μm, and stored in aliquots at -70°C.

### 2.5. Immunoglobulin concentration (Total IgG, IgG2a, and IgG2b) by ELISA

Immunoglobulin analyzes were performed on the 90th day in the control and Y groups and on the 108th day in the reinfected group (Y-Y). 96-well microtiter plates were sensitized with the crude *T. cruzi* antigen. The antigen was diluted to 5 μg/ml in carbonate bicarbonate buffer, pH 9.5. 100 μl were dispensed and these were incubated for 18 hours at 4°C. The plates were washed with PBS+0.05% Tween20 and blocked with 200 μl of PBS, containing 2% bovine serum albumin for 24 hours at 4°C. Sera diluted 1:50 for IgG, IgG2a and IgG2b and 1:250 for total IgG in PBS/2% BSA were added and incubated for 4 hours at room temperature. All plates were washed with PBS/Tween and incubated for 2 hours. Peroxidase-conjugated anti-mouse IgG antibody (Sigma-Aldrich, USA) diluted 1:25000 in PBS/2% BSA was added for the determination of total IgG. For IgG isotype analysis, anti-IgG2a and anti-IgG2b monoclonal antibodies (BD Pharmingen) were diluted 1:1000 in 1% PBS-BSA and incubated for 2 hours at room temperature. The plates were washed and re-incubated with alkaline phosphatase-conjugated Streptoavidin (BD Pharmingen) diluted 1:500 in PBS-2% BSA for 2 hours at RT. The plates were then washed again, and the assay terminated by the addition of p-nitro phenylphosphate (pNPP) in diethanolamine buffer, pH 9.2. For total IgG, Tetramethylbenzidine (TMB) was added for 30 minutes, and the reaction was interrupted with sulfuric acid to complete the assay. The dosages of IgG2a and IgG2b were incubated for 60 minutes and later read on a 405 nm filter. The total IgG dosage was read 30 minutes after the end of the assay on a 450 nm filter, and the results were expressed as absorbance.

### 2.6. Measurement of cytokines in plasma by CBA

Immunoglobulin analyzes were performed on the 90th day in the control and Y groups and on the 108th day in the reinfected group (Y-Y). The cytokines IL-4, IFN-γ (Mouse Th1/Th2) and IL-10 (Mouse Flex) were measured using the Cytometric Bead Array - CBA technique, according to the manufacturer’s instructions (BD Cytometric Bead Array - CBA). The samples and the recombinant cytokines were incubated with beads with different fluorescence intensities conjugated with a capture antibody specific to each cytokine. Then, antibodies specific to each cytokine conjugated to phycoerythrin (PE) were added. After incubation, the beads were washed with the manufacturer’s own kit solution and analyzed in the flow cytometer. The specific beads for each cytokine were separated by emitting different fluorescence. After acquiring the data from the samples and recombinant cytokines, they were taken to the FCAP Array 2.0 software (SoftFlow-USA), and the cytokine concentrations calculated from the standard curve.

### 2.7. Histological analyzes

The fragments of the intestine, descending colon, were collected, fixed in 4% formaldehyde, process and embedded in paraffin. Three sections 5 µm thick with 25 µm distance between each one was obtained and then, were staining with Hematoxylin and Eosin (HE), Giemsa and picrosirius.

To evaluate the inflammatory infiltrate, HE-stained slides were used. Each slide contained three histological sections of the intestine and a complete scan of all sections in the submucosal and muscular region was performed. The evaluation was done at a magnification of 400x. The inflammatory process was evaluated by means of semi-quantitative analysis for all groups. The intensity of the inflammatory infiltrate was classified as absent (0), when the presence of inflammatory cells was not observed, mild (1) when the inflammatory infiltrate represented 0 to 25% of the total number of cells observed, moderate (2) when the infiltrate corresponded to 26 to 50% of the cells and intense (3) if the number of inflammatory cells corresponded to more than 51% of the cells observed. After the complete evaluation of all cases, their identification was revealed. The average of the three evaluated cuts was obtained and the result for each case corresponded to a score between 0 (absent), 1 (mild), 2 (moderate) and 3 (severe).

Giemsa-stained slides were used to quantify neurons. Four serial sections with 100 μm between each were evaluated under light microscopy at 400× magnification. Neurons were counted in 30 random fields at 400× magnification in each cut (120 fields per animal). The result was expressed as the number of neurons/field.

The morphometric evaluation of collagen was performed after picrosirius staining. The slides were analyzed by digital morphometry, in a polarized light microscope, at 400x magnification. Morphometry was performed using the KS 300 Automatic Image Analyzer System – “Carl Zeiss”. Collagen was quantified in 20 random fields and the result was given as % collagen per animal.

### 2.8. Statistical analysis

Statistical analyses were performed using GraphPad Prism version 6.0 (GraphPad Software, USA). Data was analyzed using non-parametric tests, since the sample size in some experimental groups was limited and normal distribution could not be assumed. Comparisons between two groups were conducted using the Mann–Whitney test, and comparisons among three or more groups were performed using the Kruskal–Wallis test followed by Dunn’s multiple comparison test. Results are expressed as median and interquartile range (IQR), and each data point represents an individual animal. Statistical significance was considered at *p* < 0.05.

## 3. Results

### 3.1. Parasitemia

**Fig 1:**
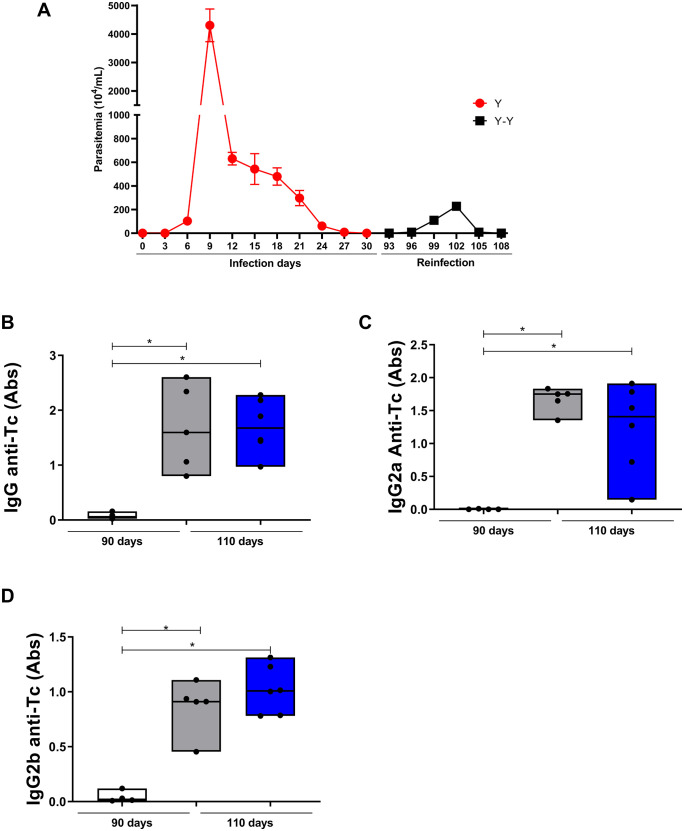
Parasitemia kinetics and antibody profile in mice infected and reinfected with *Trypanosoma cruzi* Y strain. **(A)** Parasitemia was monitored in infected (Y; red circles) and reinfected (Y–Y; black squares) C57BL/6 mice from day 0 to day 30, and again after homologous reinfection on day 90 up to day 108. Trypomastigotes were counted in fresh blood smears every three days. **(B)** Total IgG, **(C)** IgG2a, and **(D)** IgG2b levels against *T. cruzi* antigens were quantified in serum samples from uninfected mice (gray), infected mice at 90 days post-infection (dark gray; Y group), and reinfected mice at 108 days post-infection (blue; Y–Y group). Data are expressed as median and interquartile range (IQR), and individual data points represent each animal (n = 5 per group). Statistical analysis was performed using the Kruskal–Wallis test followed by Dunn’s multiple comparison test. *p* < 0.05 was considered significant.

To assess the impact of reinfection on the circulation of trypomastigotes in the blood, parasitemia was assessed both in the primary infection and in the reinfection every three days (Fig 1A). Initially, the behavior of the Y strain showed a rapid pattern of parasitemia, with the appearance of blood trypomastigotes after six days of infection, a peak at nine days and total disappearance at 30 days. After chronification of the animals (90 days), reinfection was performed and a new evaluation of parasitemia was carried out for a period of 18 days. During this period (90 to 108 days), after reinfection with the Y strain, the detection of circulating trypomastigote forms also occurred from the sixth day onwards. However, on the sixth day there was an 11-fold decrease in the number of circulating parasites. In addition, the peak was found after the 12th day of infection, differing from the primary infection in which the peak occurred on the 9th day. At this time of reinfection, there was also a significant reduction in the number of bloods trypomastigotes. Therefore, homologous reinfection results in a decreased amount of circulating trypomastigotes.

### 3.2. Levels of IgG anti-Tc antibodies

As homologous reinfection drastically reduced the levels of circulating parasites in the blood, it was then intended to assess whether the humoral immune response played an essential role in this phenomenon (Fig 1B, 1C and 1D). It was observed that the primary infection induces the production of anti-*T. cruzi* strains when compared to the uninfected group (IgG, p=0.0112; IgG2a, p=0.0036; IgG2b, p=0.0021). However, reinfection did not induce an increase in the levels of these antibodies when compared to the primary infection. However, there was a significant increase in total IgG in the group reinfected with Y (p=0.0096) when compared to the control group (Fig 1B). The same pattern was found for IgG2a immunoglobulin among the group reinfected with Y when compared to the control (p=0.0214 and p=0.0054, respectively) (Fig 1C). A similar result was found for IgG2b, with a significant increase in the group reinfected with Y (p=0.0003) when compared to the control group (Fig 1D). Therefore, the process of homologous reinfection does not influence the levels of IgG anti-Tc antiboides produced after the primary infection.

### 3.3. Production of anti-inflammatory/regulatory cytokines systemically

Given the similarity in antibody production observed between primary infection and reinfection, we next evaluated whether systemic cytokine levels could contribute to the modulation of the humoral immune response. The concentrations of IL-4, IL-10, and IFN-γ were quantified in serum samples from infected and reinfected C57BL/6 mice.

**Fig 2:**
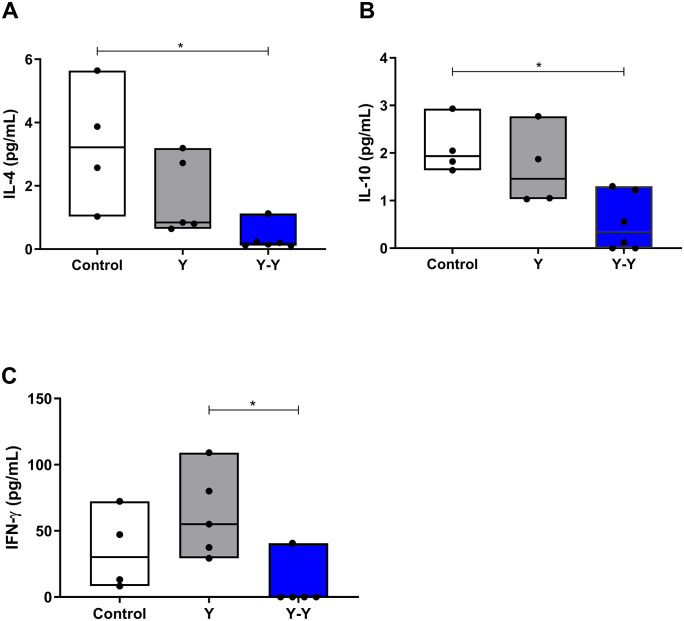
Systemic cytokine levels in mice infected and reinfected with *Trypanosoma cruzi* Y strain. Serum concentrations of **(A)** IL-4, **(B)** IL-10, and **(C)** IFN-γ were quantified using a Cytometric Bead Array (CBA) in C57BL/6 mice at 90 days post-infection (Y group) and 18 days after homologous reinfection (Y–Y group). White boxes represent uninfected control mice (*n = 5*), gray boxes represent mice infected with the Y strain (Y group; *n = 6*), and blue boxes represent reinfected mice (Y–Y group; *n = 6*). Data are expressed as median and interquartile range (IQR), and individual data points represent each animal. Statistical analysis was performed using the Kruskal–Wallis test followed by Dunn’s multiple comparison test. *p* < 0.05 was considered statistically significant.

Reinfection with the *T. cruzi* Y strain induced a significant reduction in IL-4 levels compared with uninfected controls (*p* = 0.0052; Fig 2A). Similarly, IL-10 concentrations were decreased in reinfected animals (*p* = 0.0102; Fig 2B), indicating a systemic downregulation of anti-inflammatory and regulatory cytokines. In contrast, IFN-γ levels showed a mild increase after reinfection, although without reaching statistical significance when compared with the infected group (*p* > 0.05; Fig 2C).

Together, these findings suggest that homologous reinfection with the Y strain leads to a reduction in systemic anti-inflammatory cytokines and a tendency toward a pro-inflammatory profile, consistent with the immunological activation observed after repeated exposure to the parasite.

### 3.4. Inflammatory infiltrate, neuronal quantification and collagen deposition

The control group, as expected, did not present an inflammatory process. Primarily infected animals showed a mild inflammatory infiltrate in 80% of the animals (4/5 animals) and moderate in 20% (1/5 animals). In the group reinfected with the Y strain, a mild inflammatory cell infiltrate was observed in 60% (3/5 animals) of the mice and moderate in 40% (2/5 animals).

**Fig 3:**
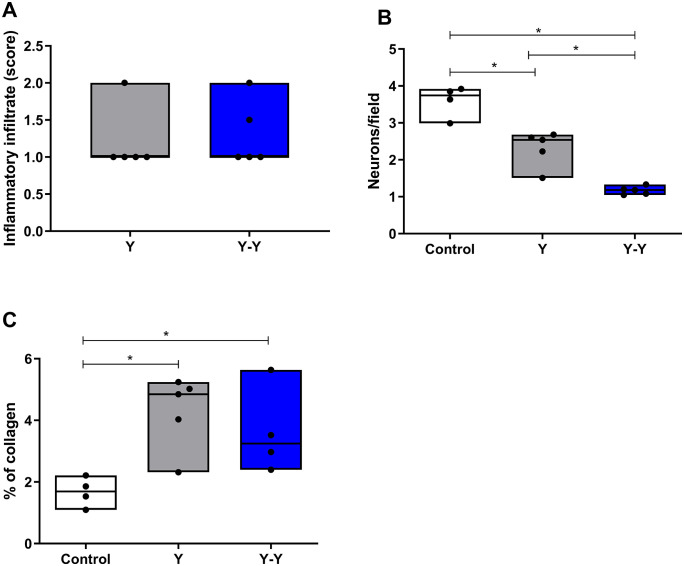
Intestinal inflammation, neuronal loss, and collagen deposition in mice infected and reinfected with *Trypanosoma cruzi* Y strain. Histopathological analysis of intestinal tissue from C57BL/6 mice at 90 days post-infection (Y group) or 18 days after homologous reinfection (Y–Y group). **(A)** The inflammatory infiltrate was scored semi-quantitatively in the submucosa and muscular layers. **(B)** The number of neurons per field in the myenteric plexus was determined using Giemsa staining. **(C)** The percentage of collagen deposition in the intestinal wall was quantified by picrosirius-red staining under polarized-light microscopy. White boxes represent uninfected control mice (*n = 5*), gray boxes represent mice infected with the Y strain (Y group; *n = 6*), and blue boxes represent reinfected mice (Y–Y group; *n = 6*). Data are expressed as median and interquartile range (IQR), and each dot represents an individual animal. Statistical analysis was performed using the Kruskal–Wallis test followed by Dunn’s multiple-comparison test. *p* < 0.05 was considered statistically significant.

The assessment of neuronal loss was performed using Giemsa staining in all experimental groups (Fig 3B). It was observed that the primary infection was able to induce neuronal destruction (p=0.0159). Interestingly, reinfection can intensify the neuronal destruction previously observed in the primary infection (Y-Y, p=0.0079)

**Fig 4:**
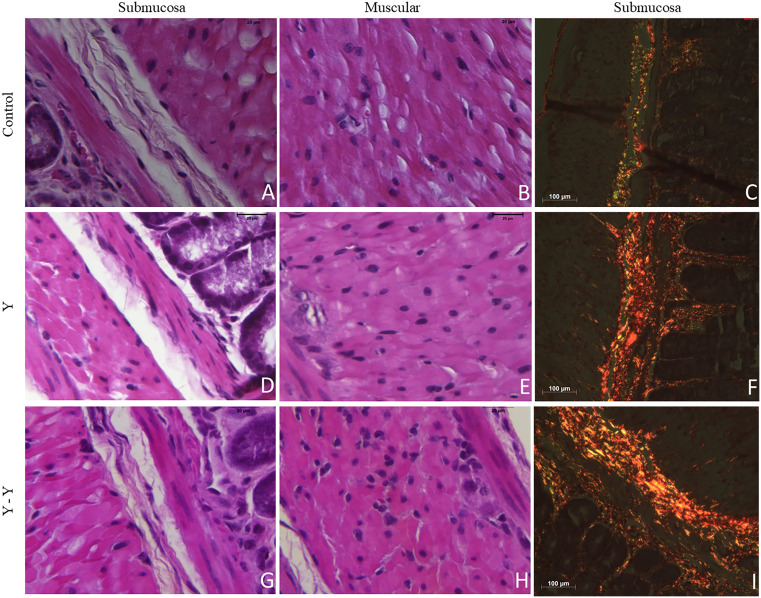
Histological sections showing intestinal inflammation and collagen deposition in infected and reinfected mice. Representative photomicrographs of intestinal tissue from C57BL/6 mice. Hematoxylin and eosin (HE) staining of the submucosal and muscular layers shows inflammatory infiltrate in: **(A, B)** control group; **(D, E)** mice infected with *T. cruzi* Y strain; **(G, H)** mice infected and reinfected with the Y strain. Picrosirius staining under polarized light shows collagen deposition in the submucosal layer in: **(C)** control group; **(F)** infected group; **(I)** infected and reinfected group.

Morphologically, the presence of inflammatory infiltrates across the experimental groups is evident in both the submucosal and muscular layers of the intestine, as illustrated in Fig 4 (panels A, B, D, E, G and H).

Regarding collagen deposition, both the primary infection and reinfection groups exhibited increased collagen accumulation when compared to the uninfected control group (p = 0.0159 and p = 0.0286, respectively; Fig 3C). This increase is also visually apparent in Fig 4 (panels C, F and I).

Thus, regardless of the degree of inflammatory infiltrate, reinfection appears to intensify the neuronal destruction initially triggered by the primary infection.

## 4. Discussion

Data regarding the impact of reinfection in *T. cruzi* infection remains contradictory. Some studies suggest that repeated exposures may enhance immune responses and progressively reduce parasitemia. For instance, Machado *et al.* (2001) demonstrated that successive reinfections in dogs led to decreased parasitemia and the development of the indeterminate form of Chagas disease, without mortality [27]. In contrast, other reports indicate that reinfection can be associated with increased mortality [10,23]. In our study, primary infection with the Y strain appeared to confer protection against homologous reinfection, as evidenced by reduced levels of circulating parasites.

To better understand the mechanisms behind this protection, we evaluated humoral and systemic cytokine responses. Notably, reinfection did not lead to an increase in total IgG, IgG2a, or IgG2b levels, yet parasitemia was significantly reduced in the reinfected group. This suggests that circulating antibodies generated during the primary infection remained effective in controlling the parasite upon reinfection, potentially in conjunction with other immune mechanisms. Previous studies have also reported high IgG production during the acute phase and persistence into the chronic phase of Chagas disease [31,32], which could explain the parasitemia control observed in our model.

Among IgG subclasses, IgG2a is known to contribute to parasite clearance [33], while IgG2b has been classically described as protective against *T. cruzi* [34,35]. In our study, IgG2b levels were higher in the reinfected group, suggesting a possible role in the improved control of parasitemia. Conversely, IgG2a levels were more elevated in the infected group, which exhibited greater parasitemia. These findings may reflect different immunoglobulin kinetics or functionalities and merit further investigation to clarify their respective protective roles.

Regarding cytokine profiles, reinfection was associated with reduced systemic levels of IL-4 (anti-inflammatory) and IL-10 (regulatory), reinforcing the notion that the immunopathological processes observed in the acute phase persist during reinfection. Although humoral immunity appears to control parasitemia, it does not fully prevent tissue damage. Inflammatory responses with a pro-inflammatory cytokine profile seem to contribute to neuronal destruction in the intestinal wall. We did not observe an increase in collagen deposition, likely due to the short time interval between reinfection and tissue analysis.

IL-4 and IL-10 are associated with the inhibition of Th1 responses and excessive inflammation, which, when unregulated, can cause tissue damage [36,37]. In human Chagas disease, a more pro-inflammatory profile is correlated with severe cardiac and digestive forms, whereas anti-inflammatory responses are more common in asymptomatic cases [38–40]. Moreover, studies in human megacolon and megaesophagus have demonstrated a strong correlation between local inflammation, nitric oxide, IFN-γ, TNF-α, and neuronal destruction [41,42], which begins during the acute phase and progresses with chronicity [28].

Despite the relevance of our findings, a limitation must be acknowledged: the use of a single *T. cruzi* strain (Y) for both infection and reinfection. While this choice ensured experimental consistency, it does not replicate the heterologous reinfection scenarios common in endemic areas. Future studies employing different *T. cruzi* strains could provide additional insights into the dynamics of reinfection and its implications for disease progression.

## 5. Conclusion

Reinfection with the Y strain of *Trypanosoma cruzi* in this experimental model aggravated the course of intestinal Chagas disease, leading to more intense tissue inflammation, increased collagen deposition, and significant neuronal loss in the myenteric plexus. Although the heightened production of IgG2a and IgG2b subclasses appeared to contribute to parasitemia control, these immunoglobulins were insufficient to restrain the progression of local inflammatory damage. The immune milieu observed marked by a predominance of pro-inflammatory cytokines such as IFN-γ over regulatory (IL-10) and anti-inflammatory (IL-4) mediators suggests that reinfection amplifies immunopathological processes in the intestinal tract.

These findings highlight the dual role of the immune response in chronic Chagas disease: while essential for parasite containment, excessive or dysregulated inflammation may accelerate tissue injury and neuronal depletion. Nevertheless, it is important to consider that host–parasite interactions, particularly the combination of the C57BL/6 mouse strain and the Y lineage of *T. cruzi*, may influence disease outcomes and immune polarization, limiting the extrapolation of these results to other experimental systems. Furthermore, the absence of cellular-level analyses such as T-cell subset characterization and cytokine production profiling, represents an additional limitation that should be addressed in future studies.

Taken together, the present data reinforce that repeated exposure to *T. cruzi* can intensify intestinal pathology through sustained pro-inflammatory activity, underscoring the importance of controlling reinfection in endemic areas. Expanding these findings to include different host–parasite models and cellular immune parameters will be essential to better elucidate the mechanisms driving chronic intestinal damage and to inform strategies aimed at mitigating the long-term consequences of Chagas disease.
